# Effects of incubator oxygen and carbon dioxide concentrations on hatchability of fertile eggs, some blood parameters, and histopathological changes of broilers with different parental stock ages in high altitude

**DOI:** 10.1016/j.psj.2021.101609

**Published:** 2021-11-19

**Authors:** Nezih Okur, Sabri Arda Eratalar, Ayşe Arzu Yiğit, Tuncer Kutlu, Ruhi Kabakçi, Şule Yurdagül Özsoy

**Affiliations:** ⁎ Department of Poultry Science, Faculty of Agriculture, Bolu Abant İzzet Baysal University, Bolu, Turkey; † Department of Physiology, Faculty of Medicine, Başkent University, Ankara, Turkey; ‡ Department of Pathology, Faculty of Veterinary Medicine, Hatay Mustafa Kemal University, Hatay, Turkey; § Department of Physiology, Faculty of Veterinary Medicine, Kırıkkale University, Kırıkkale, Turkey; ‖ Department of Pathology, Faculty of Veterinary Medicine, Aydın Adnan Menderes University, Aydın, Turkey

**Keywords:** broiler, carbon dioxide, incubation, oxygen, parental stock age

## Abstract

The effects of incubator carbon dioxide (**CO_2_**) and oxygen (**O_2_**) concentrations with parental stock age (**PSA**) on embryonic deaths (**ED**), hatchability of fertile eggs (**HFE**), some blood parameters, and the tissue development of broilers were investigated. Four consecutive repetitions following the similar materials and methods were carried. From 3 different aged ROSS 308 broiler parental flocks 7,680 hatching eggs were obtained and classified as young (Y; 29 wk), middle (M; 37 wk) and old (O; 55 wk) as regards PSA, and randomly distributed. Four different incubator ventilation programs (**IVP**) as control (**C**; 0.67% CO_2_ and 20.33% O_2_), high CO_2_ (**HC**; 1.57% CO_2_ and 20.26% O_2_), high O_2_ (**HO**; 0.50% CO_2_ and 21.16% O_2_), and high CO_2_ + O_2_ (**HCO**; 1.17% CO_2_ 21.03% O_2_) were applied with oxygen concentrator, and ED and HFE were investigated. Lung and heart tissues, hemoglobin value, packed cell volume, and red blood cell count, triiodothyronine, thyroxine, adrenocorticotropic hormone (**ACTH**) values of the chicks were analyzed. It was found that IVP affected ED and HFE. Higher rate of early ED (**EED**) was obtained from the HC than HCO, and higher middle+late stage+pipped but unhatched ED (**MLPED**) with a lower rate of HFE was observed in the C group than HO and HCO (*P* < 0.05). Association was found between PSA and IVP (*P* < 0.05), being more evident in EED for young PSA, in MLPED with HFE for Y and O PSA. From hematological values, no statistical difference in RBC, PCV, and Hb values were found among the treatment groups, ACTH concentration known as a response to stress was found to be higher than C in all groups, triiodothyronine concentration was higher in the HO group than C. In the histopathological examination, used IVPs were found to have negative effects on the lung and heart such as vacuolization, hemorrhage in all PSA groups except for C. Conclusively, PSA and IVP affected some hatching, blood and tissue development parameters of the broiler chicks.

## INTRODUCTION

Natural incubation is a dynamic and magnificent process, resulting in optimum hatchability of fertile eggs (**HFE**) and chick quality. This process can be imitated by artificial incubation that requires an optimum counterpoise between some important factors in order to achieve the best HFE. These factors include pre-incubation conditions such as parental stock age (**PSA**), egg weight (**EW**), eggshell conductance, and environmental factors during incubation such as temperature, humidity, gas concentrations, and altitude (Visschedijk 1991; Hassanzadeh et al., 2004; De Smit et al., 2006; Elibol and Brake, 2008; Meijerhof, 2009).

Parental stock age is a parameter known to affect EW, fertility (**F**), HFE, tissue development in broilers. As PSA increases, EW also increases (Suarez et al., 1997; Huwaida et al., 2015), while H and fertility rate decrease (Suarez et al., 1997). Furthermore, the range of the early embryonic deaths (**EED**), and mid and late stage+pipped but unhatched embryonic deaths (**MLPED**) change according to PSA (Tullett, 2009).

Gas exchanges during incubation are vital in embryonic development having possible effects on hatching performance and chick quality. To achieve normal embryonic development adequate oxygen levels and removal of sufficient carbon dioxide is needed (Ar and Deeming, 2009). Inadequate aeration, lack of air movement or provision of fresh air may cause hypoxic and hypercapnic conditions. Those have significant adverse effects on embryonic deaths and survivability of incubated birds (Mersten-Katz et al., 2013) and their embryos (Tullett and Deeming, 1982). In the field, chicken hatching eggs are incubated in an environment of 0.5% carbon dioxide (**CO_2_**) and 21% oxygen (**O_2_**) gases (De Smit et al., 2006). It is known that atmospheric air contains 0.3 to 0.4% CO_2_ and about 21% O_2_. However, the CO_2_ air concentration can exceed 1%in the nest during natural incubation. Simultaneously O_2_ concentration in the egg's air cell decrease below 17% soon before hatch (Visschedijk, 1968; Deeming and Ferguson, 1991). In addition, it is known that actual broiler strains are bred for fast growth and high feed efficiency, and therefore, more O_2_ is needed to meet these hybrid birds’ high metabolism (Fernandes et al., 2014). Some recent studies imply that hatching and field performance are negatively affected by both low (<17%) and high (25%) O_2_ concentrations (Stock and Metcalfe, 1984; Lourens et al., 2007; Molenaar et al., 2010).

It is known that O_2_ consumption and CO_2_ production increase as the embryo develops (Decuypere et al., 2001). The demand for oxygen and tolerability of the embryo is lowest in the first 5 d and both tolerances increase during incubation (Taylor et al., 1971; Everaert et al., 2007). As the altitude increases, the partial pressure of O_2_ reduces which influences the gas exchanges of the egg (Visschedijk, 1991). In addition, lung capacity cannot always meet the oxygen requirement for the fast-growing broilers, and hypoxemia and ascites can occur especially in ascites susceptible strains (Beker et al., 1995; Currie, 1999; Julian, 2000).

Oxygen and, accordingly CO_2_ concentrations are known to affect embryonic deaths (**ED**) and HFE (De Smit et al., 2006). Several industrial incubator ventilation programs allow higher CO_2_ concentrations up to 1.5% in the first 10 days of incubation to stimulate embryonic development, better H, HFE, and hatching synchronization (Decuypere et al., 2006; De Smit et al., 2006). De Smit et al. (2006) implied increasing CO_2_ concentrations at this period improved H and HFE in 60 wk old breeders. Jozsa et al. (1986) determined that around the 14th day of embryonic development, Corticotropin releasing hormone (**CRH**) release from hypothalamus occurred, and around the 16th d, adrenal growth and development began by adrenocorticotropic hormone (**ACTH**) stimulation regulated by CRH. Increased CO_2_ concentration towards the end of incubation is a stimulus for increasing plasma corticosterone, stimulating ACTH, and increasing tyroide hormones such as triiodothyronine (**T_3_**) and thyroxine (**T_4_**) leading the onset of hatching of the chicks (Decuypere et al., 2006). Similarly, Blacker et al. (2004) reported corticosterone level was increased under hypoxic stress. Furthermore, Decuypere et al. (2006) and De Smit et al. (2006) determined that T_4_ regulates the interval between pulmonary respiration and hatching.

Hassanzadeh et al. (2004) indicated that the T_3_, T_4_, and corticosterone concentrations of chick embryos incubated at a high altitude were higher than those incubated at a low altitude. Similar results were found by Sahan et al. (2011) who reported that while plasma T_3_ concentration was higher, plasma T_4_ concentration did not change at higher altitudes in hatched chicks. Sahan et al. (2011) also showed that O_2_ supplementation to the hatcher in high altitudes did not change T_3_ and T_4_ concentrations as compared to the control group.

It is known that as the altitude increases, the atmospheric pressure decreases, so at the same time the partial pressure (mm Hg or Pa) of individual air components, but its percentage composition does not change. However, contrary to blood parameters, the development of various respiratory organs such as heart and lung changed depending on the O_2_ concentration in incubator in accordance with the altitude (Maxwell et al., 1987; Beker et al., 1995). In order to benefit from the insufficient O_2_, hypertrophy is observed, developing abnormal, and overgrown organs (Julian, 2000; Santos et al., 2005). Right ventricle weights were higher and lung and liver weights were lower under low O_2_ (13%) when compared with normal (20.6%) conditions.

Therefore, it is necessary to compensate for the oxygen content or increase the pressure in the incubator in high altitudes. In addition, in places with high altitudes like India and South America (3,500–4,000 m), very poor H rates (e.g., 20%) have been found (Ahmed et al., 2013). To untangle this distress, supplementary systems that increase O_2_ concentration in incubators are widely used. It was suggested that O_2_ concentration in the incubator should be augmented by 8.5% in areas higher than 750 m (Cobb, 2013) and 13.8% with (˃1,500 m) higher altitudes (Tullett, 2013). Furthermore, Sahan et al. (2011) reported that survival rate increased and late stage ED and Hb value decreased while PCV, T_3_, and T_4_ values did not change in high O_2_ supplementation to the hatcher at altitudes of 1,100 m.

Some conflicting data were found in studies that investigated effects of CO_2_ and O_2_ on incubation performance (Onagbesan et al., 2007; Piestun et al., 2008). High CO_2_ during incubation was also found to have different effects on incubation and post-hatch performance (Everaert et al., 2007; Fernandes et al., 2014; Ozlu et al., 2019), according as the concentration of exposure, time, and duration of the CO_2_ application (Tona et al., 2007; Maatjens et al., 2014a,b; Tong et al., 2015). Though, these effects are known to differ between broiler lines (De Smit et al., 2008; Tona et al., 2013).

The aim of this study was to evaluate whether different O_2_/CO_2_ concentrations affect HFE and the survival of the embryo at the altitude of 822 m. Additionally, it was aimed to examine if the manipulation had any effects on hematologic parameters and the corticosterone and thyroid hormones. Furthermore, lung and heart histopathology were reviewed.

## MATERIALS AND METHODS

Animal Welfare Legislation of Turkey was carried out during the trial and all procedures during handling of the eggs and chicks were approved by the Animal Ethics Committee of Bolu Abant Izzet Baysal University (Decision No: 2018/20).

### Biologic Materials and Equipment Used During the Trial

The study consisted of 4 similar trials repeated 4 times following the same materials and methods.

In each trial, 1,950 and 7,800 hatching eggs in total were taken from 3 different ROSS 308 broiler parental flocks (young/29 wk, middle/37 wk and old/55 wk) from a commercial company in Bolu/Turkey. Thus, the number of eggs for each PSA group was 650 in each trial. Ten dingy, fractured, wrinkled and thin-shelled eggs from each PSA group were discarded for being unsuitable. Remaining 1,920 hatching eggs were weighed (±0.1 mg) by an analytical balance (Radwag AS 220.R2, Radwag Balance and Scales, Poland) and numbered individually and recorded for each group.

Incubation process was carried out in Bolu Abant Izzet Baysal University's Faculty of Agriculture Department of Poultry Science incubation laboratory with 4 duplicate incubators with a capacity of 480 eggs each (Cimuka 960SH, Cimuka Ltd. Co., Turkey). The whole incubation process took place in these incubators, each equipped with 6 trays carrying 80 eggs, and 6 hatch baskets with the same capacity. In addition, 2 oxygen concentrators (Hikoneb Oxybreath 10LPM, Kare Medical, Ltd. Co., Turkey) were used to increase the oxygen content of the air supplied into 2 of the incubators.

### Trial Design

A lay-out plan for the eggs was prepared to provide eggs with similar weights (same PSA) should be set in the egg trays, in the same incubator and treatment group. After this they were placed in their appointed positions according to the lay-out plan and randomly distributed to a total of 6 trays in the incubators and 4 incubators operated according to the 4 different ventilation programs (Table 1).

**Table 1:** The number of eggs used in the experiment and their distribution according to the experimental groups in each incubator.

	Parental stock age (PSA)			
	Young (Y)	Middle (M)	Old (O)	Total
Start up	650	650	650	1,950
Selection	10	10	10	30
After selection	640	640	640	1,920
1st tray	27	26	27	80
2nd tray	27	27	26	80
3rd tray	26	27	27	80
4th tray	27	26	27	80
5th tray	27	27	26	80
6th tray	26	27	27	80
Total per incubator / IVP	160	160	160	480

Abbreviations: PSA, parental stock age, Y, young (29 wk), M, middle (37wk), O, old (55 wk); IVP, incubator ventilation program C, control (0.67% CO_2_ and 20.33% O_2_), HC, high CO_2_ (1.57% CO_2_ and 20.26% O_2_), HO, high O_2_ (0.50% CO_2_ and 21.16% O_2_), HCO, high CO_2_ + O_2_ (1.17% CO_2_ 21.03% O_2_).

It is known that at normal air pressure and oxygen partial pressure at an altitude of zero meter above sea level, while at a higher altitude, air pressure, and oxygen partial pressure will different. Therefore, at high altitudes, to ensure the same oxygen availability as at sea level, either increase the pressure in the room (by using a kind of hyperbaric chamber) or artificially enrich the air with oxygen (e.g., from a cylinder).

However, the experiment was organized based on the idea of field applications and ease of application in the field instead of sensitive laboratory conditions, and treatment groups were tried to be formed according to the options that can be applied in incubation conditions. Consequently, it is known that O_2_ concentration should be increased by about 8.5% in places with 750 m or higher altitude (Cobb, 2013; Tullett, 2013). The experiment took place at an altitude of 822 m at the coordinates of 40°42′53.62′′ N, 31°31′29.82′′ E. It was reported that increasing the CO_2_ concentration to about 1.0 to 1.5% at 10th d of incubation improves H (Tona et al., 2007; Tong et al., 2015), varying in concur with chicken lines (De Smit et al., 2008; Tona et al., 2013). Regarding this information, the incubation test trials were performed before the experiment, closing the main air inlets and the change in CO_2_ and O_2_ concentrations in the machine were monitored and recorded. In the test trials, during first 10 days of incubation, CO_2_ concentrations increased up to 1.58% and O_2_ concentrations decreased to 20.26%. Just after air inlets were opened, CO_2_ concentrations decreased to 0.7% and O_2_ concentrations increased up to 21.40% by using oxygen concentrator to enrich the air into incubator. Regarding these results of the test trials done and from the findings of the literature mentioned above, incubator ventilation programs (**IVP**) treatment groups were formed for the main experiment.

The treatment groups were formed as young (Y), middle aged (M) and old (O) for PSA; and control (C), high O_2_ (HO), high CO_2_ (HC), and high CO_2_ + high O_2_ (HCO) for the IVP. For each group, a single tray was taken as a replicate and a total of 6 replicates was used (Table 1).

### Incubation Period

Hatching eggs were stored for 3 d prior to incubation and the storage room temperature was kept at 18°C and the humidity at around 75%. After the storage period, setter trolleys with pre-set egg trays were randomly placed in the 4 identical incubators. Before the incubation period, the incubators were kept at 24°C for 6 h to preheat the eggs.

Setter trays were placed and the incubation process was started. To get eggshell temperature (**EST**) data correctly 2 eggs from each tray with a total of 12 for each incubator were measured twice a day (9:00 and 17:00) by an infrared ear thermometer (Braun Thermoscan 7 IRT6520, Braun GmbH, Deutschland) and were recorded and utilized if needed to provide the optimal incubator settings. Mean EST values were calculated, and in case of any deviation from the expected temperature, the incubators’ temperature settings were immediately adjusted as required. During the incubation period, all incubators were operated to achieve an EST of 37.78°C (100.0°F).

Relative humidity in incubators was maintained as 57.5% until transfer, then increased to 58.0% during the transfer, to 60.0% during pipping and to 70.0% during hatching. Also, the humidity of the incubation room was adjusted to 50.0% using 2 cold and warm humidifiers (Weewell WHC752, Foshan Samyo Electronic Co. ltd., China) to make sure that the machine humidity was kept at 57% easily and not fluctuate. Egg weight loss is checked separately in each tray during transfer and the average weight loss on the 18th d during the transfer was 12%. Egg-turning was done (hourly) 24 times/day.

First incubator was taken as control group (**C**) and no IVP treatment was applied. The second incubator was assigned as the HO group and the ventilation system was not altered except for the provision of high O_2_ concentration into the incubator with an oxygen generator and increasing concentrations of O_2_. The O_2_ pureness of the oxygenated air was 92.0% ± 3.0. The oxygen content was assumed to be about 10% lower than normal conditions under trial conditions, and the amount of O_2_ supplied was adjusted accordingly. Remaining 2 incubators’ air inlets were shut and CO_2_ was let to increase during the first 10 days of incubation (HC and HCO groups). Right after air inlets were opened, and the normal ventilation program was carried out. Air with enriched O_2_ concentration was blown into one of these (high CO_2_ + O_2_, HCO) by an oxygen generator identical to the one used in HO group.

The O_2_ concentrations in incubators were daily recorded by O_2_ data-loggers (PAC 7000, Dragger Safety AG&Co. KGaA, Deutschland), the CO_2_ levels by incubators’ sensors (Hatch Eco 2-01, Çimuka Ltd. Co., Turkey), incubator temperature and humidity incubators’ standard sensors (KPL215, Galtech+Mela GmbH, Germany). Thus, IVP treatment groups to incubators were control (C: 0.67% CO_2_ and 20.33% O_2_), high CO_2_ (HC: 1.57% CO_2_ and 20.26% O_2_), high O_2_ (HO: 0.50% CO_2_ and 21.16% O_2_), and high CO_2_ + O_2_ (HCO: 1.17% CO_2_ 21.03% O_2_) in the experiment, at 0.67% / 20.33%, 1.57% / 20.26%, 0.50% / 21.16% or 1.17% / 21.03% during incubation in the incubators.

At 18th d, eggs were transferred from trays to the hatch baskets with the same lay-out. After completion of hatches, embryonic deaths were determined as early stage (0–5th days as EED), middle stage (6–17th), and late stage (18–21th d) + pipped but unhatched (MLPED), then true fertility (**F**) and using these data H values were calculated.

### Blood and Tissue Analysis

Fifteen newly hatched chicks from each group were killed by cervical dislocation and the collected blood samples in EDTA were promptly used for RBC, PCV, and Hb analysis. The rest of the blood was centrifuged at 2,500 rpm at 10 min to obtain plasma samples which were stored at −20 C until T_3_, T_4_, and ACTH analysis. While collecting blood, the lung and heart were also taken for histopathologic examination. Plasma ACTH, T_3_, and T_4_ concentrations were measured only in the hatched chicks from middle-aged parental flocks due to the highest F and H, as indicated in Table 3.

For RBC count, blood was diluted with a Natt-Herrick solution 200 times and counted on a Thoma hemocytometer. Erythrocytes located in the 4 corners and the central squares were counted in each chamber. Mean number was calculated and multiplied by dilution factor. Total Hb concentration was determined using the cyanmethemoglobin method. PCV was measured by microhematocrit tubes after microhematocrit centrifugation at 13,000 rpm in 5 min (Campbell and Ellis, 2007).

Hormone analysis was performed by chicken T_3_ (Cusabio; Catalog Number. CSB-E13270C), chicken T_4_ (Catalog Number; CSB-E15787C), and chicken ACTH (Catalog Number; CSB-E14373C) ELISA kits.

### Histopathologic Analysis

Lung and heart tissues were fixed in 10% buffered formalin. Then, 5-μm thick sections were cut from the paraffin-embedded blocks after a series of alcohol and xylol treatments, deparaffinized in xylol, stained with Hematoxylin and Eosin after passing through a series of 100, 96, 80, and 70% alcohol treatments. Microphotographs (Olympus DP12) were obtained after the examination under a light microscope (Olympus CX31). Histopathological findings were graded as none (−), very mild (±), mild (+), moderate (++), and severe (+++).

### Statistical Analysis

The factors: PSA and IVP were used in a 3 × 4 factorial arrangement. The quantity of replicates was calculated by power analysis with PASS 11 (Hintze, 2011) and the replication quantity for each group was determined as 6. The results were based on actual data since the differences were not significant (*P* > 0.05) between the data with and without arc sinus transformation. The statistical analyses of the results were performed using IBM SPSS 22 (SPSS, 2013), by first obtaining the skewness and kurtosis values, and then confirming the normal distribution by Shapiro-Wilk test. After this process, an analysis of variance (**ANOVA**) was undertaken for the experiment using the GLM procedure of SPSS (2013) appropriate for 2-way designs. The 2-way ANOVA model is as follows:

$$Y_{\text{ijk}}=\mu +\text{PS}A_{i}+\text{IV}P_{j}+{\left(\text{PSAIVP}\right)}_{\text{ij}}+e_{\text{ijk}}$$

where Y_ijk_ is the dependent variable, μ is the overall mean, PSA_i_ is the effect of PSA (i = young, middle or old in the experiment), IVP_j_ is the effect of the IVP used (j = control (C), high CO_2_ (HC), high O_2_ (HO) and high CO_2_ + O_2_ (HCO) in the experiment), PSAIVP_ij_ is the effect of the interaction between PSA and IVP, and e_ijk_ is the random error term. The 2-way ANOVA and post-hoc Tukey test were used to analyze the differences in the investigated parameters in relation to PSA and IVP, as well as their interaction (Kocabas et al., 2013).

The statistical analysis of the RBC count, Hb and PCV values, T_3_, T_4_, and ACTH concentrations were carried out using GraphPad Prism 6.0 (GraphPad Software, La Jolla, CA) for Windows. All data were assessed by one-way ANOVA analysis with Tukey's post-hoc test to compare the means of each series of experiments. *P*-values of less than 0.05 were considered as statistically significant. All the data were given as means ± standard error of the means (M ± SEM).

## RESULTS AND DISCUSSION

O_2_ and CO_2_ exchange is vital for the embryonic cells. If the eggs are stored in a closed environment, gas exchange related problems affect egg quality, H, pipping, and the development of the embryo (Decupeyre et al., 2001). In modern hatcheries, chicken eggs are incubated in the presence of 21% O_2_. Due to the increased metabolic activities in the second half of the incubation, CO_2_ rate increases and the growing embryo need more O_2_ (Stock and Metcalfe, 1987). Incubators are designed to provide O_2_ to the embryos and exhaust excessive CO_2_ from the machine (Onagbesan et al., 2007). The quality of the O_2_ varies with altitude. It decreases with high altitude which affects the incubation period and H (Visschedjik, 1991; Hassanzadeh et al., 2004).

Late hypoxia or hypercapnia may be beneficial in lowering the occurrence rate of ascites during the growth period of broilers. It is suggested that increased CO_2_ during the first 10 days of incubation (early hypoxia or hypercapnia) may result in an increased sensitivity for ascites-inducing factors (De Smit et al., 2006). According to Altan et al. (2006), RBC Hb, and PCV values in the control group and the oxygen supplemented group did not change. However, lower late stage ED and accordingly higher H were obtained (Maxwell et al, 1995; Altan et al., 2006). Furthermore, erythrocyte, leukocyte, PCV, and Hb values did not change in low O_2_ concentrations in the study conducted by Beker et al. (1995). Additionally, Tong et al. (2015) reported that hypercapnia had no effect of on the PCV and Hb values.

The results of this experiment, which was planned based on conditions like the field application and solution suggestions against possible problems, were evaluated and discussed in the light of this information.

### PSA

The egg weights of the PSA groups (young, middle-aged, and old) were 56.19 ± 0.09 g (CV_EW_ = 8.11), 62.17 ± 0.08 g (CV_EW_ = 6.11), 67.61 ± 0.10 g (CV_EW_ = 7.34), respectively in this study. Young (Y), middle-aged (M), and old (O) broiler parental flocks’ EED (2.06, 1.72, and 2.67%, respectively) and MLPED (3.42, 3.55 and 5.25% respectively) were lower than specs (5.50, 3.50, and 8.00% for EED; 6.00, 7.05 and 9.50% for MLPED, respectively; Tullett, 2009). It was found that PSA did affect EED and MLPED and the results were in line with the previous studies (Abiola et al., 2008) that reported an increase in EED and MLPED with increased age in older flocks.

The hatchability of fertile eggs rates in O was lower in comparison to Y and M (*P* < 0.05, Table 2). This was a result of increased MLPED values (*P* < 0.05).

**Table 2:** The effects of parental stock age, incubator ventilation program on broilers’ embryonic deaths and hatchability (M ± SEM).

		Embriyonic deaths, %			
	Fertility %	Early stage 0–5 d	Middle and last stage + pipped but unhatched	Hatchability of fertile eggs %	Hatchability %
Main effects					
Parental stock age, BA, week					
Young, Y, 29	91.90 ± 0.67^b^	2.06 ± 0.30	3.42 ± 0.44^b^	94.76 ± 0.44^a^	86.66 ± 0.76^b^
Middle, M, 37	96.48 ± 0.39^a^	1.72 ± 0.28	3.55 ± 0.38^b^	94.95 ± 0.39^a^	91.44 ± 0.49^a^
Old, O, 55	76.56 ± 1.33^c^	2.67 ± 0.47	5.25 ± 0.45^a^	92.78 ± 0.53^b^	69.34 ± 1.37^c^
Incubator Ventilation Program, IVP					
Control, C	89.64 ± 1.33	1.72 ± 0.29^b^	5.37 ± 0.62^a^	92.88 ± 0.64^b^	82.52 ± 1.55
High CO_2_, HC	90.27 ± 1.43	3.26 ± 0.60^a^	4.49 ± 0.42^ab^	93.76 ± 0.53^ab^	84.04 ± 1.65
High O_2_, HO	86.69 ± 1.56	1.80 ± 0.33^ab^	3.37 ± 0.36^b^	94.82 ± 041^ab^	81.51 ± 1.66
High CO_2_+O_2_, HCO	86.65 ± 1.64	1.75 ± 0.35^b^	3.06 ± 0.39^b^	95.19 ± 0.53^a^	81.85 ± 1.71
Interaction, BA × IVP					
YC	92.90 ± 1.34^a^	1.69 ± 0.43^b^	4.86 ± 0.90^a^	93.45 ± 0.93^ab^	86.35 ± 1.50^a^
YHC	94.94 ± 0.99^a^	2.64 ± 0.67^ab^	3.95 ± 0.69^ab^	94.35 ± 0.89^ab^	89.29 ± 1.34^a^
YHO	89.14 ± 1.63^a^	1.67 ± 0.63^b^	2.62 ± 0.55^b^	95.71 ± 0.82^a^	84.84 ± 1.84^a^
YHCO	90.63 ± 1.41^a^	2.23 ± 0.63^ab^	2.24 ± 0.57^b^	95.53 ± 0.83^a^	86.17 ± 1.25^a^
MC	96.81 ± 0.56^a^	1.67 ± 0.57^b^	3.36 ± 0.97^ab^	94.97 ± 0.98^a^	91.78 ± 1.13^a^
MHC	96.64 ± 0.86^a^	1.87 ± 0.58^b^	4.29 ± 0.73^b^	94.76 ± 0.79^a^	91.40 ± 0.90^a^
MHO	96.42 ± 0.80^a^	1.86 ± 0.57^b^	3.57 ± 0.58^b^	94.57 ± 0.50^a^	90.99 ± 1.03^a^
MHCO	96.07 ± 0.88^a^	1.50 ± 0.57^b^	2.99 ± 0.74^b^	95.51 ± 0.84^a^	91.57 ± 0.94^a^
OC	79.22 ± 2.31^b^	1.88 ± 0.51^b^	7.90 ± 1.10^a^	90.22 ± 1.19^b^	69.44 ± 2.16^b^
OHC	79.24 ± 2.75^b^	5.42 ± 1.50^a^	5.22 ± 0.78^ab^	92.17 ± 1.00^ab^	71.41 ± 3.19^b^
OHO	74.51 ± 2.53^b^	1.88 ± 0.51^b^	3.93 ± 0.74^b^	94.19 ± 0.74^ab^	68.70 ± 2.70^b^
OHCO	73.26 ± 2.90^b^	1.50 ± 0.63^b^	3.96 ± 0.70^b^	94.54 ± 1.07^a^	67.80 ± 2.92^b^
*P* values					
PSA	0.000	0.171	0.002	0.001	0.000
IVP	0.181	0.019	0.001	0.010	0.708
PSA × IVP	0.000	0.009	0.000	0.001	0.000

Abbreviations: IVP, incubator ventilation program, C, control (0.67% CO_2_ and 20.33% O_2_), HC, high CO_2_ (1.57% CO_2_ and 20.26% O_2_), HO, high O_2_ (0.50% CO_2_ and 21.16% O_2_), HCO, high CO_2_ + O_2_ (1.17% CO_2_ 21.03% O_2_).

abc Different superscript letters show that difference between means of groups are statistically significant (*P* < 0.05).

The HFE of the PSA group was found to be lower slightly different than specs (94.76–90.43 in Y, 94.95–93.55 in M and 92.78–85.76 in O, respectively) (Tullett, 2009; Aviagen, 2016). This is thought to be due to improved breeding, management, and feeding conditions. It was found that PSA affected HFE causing HFE to decrease with increasing flock age in older flocks (Suarez et al., 1997; Elibol and Brake, 2008).

### IVP

**Figure 1:**
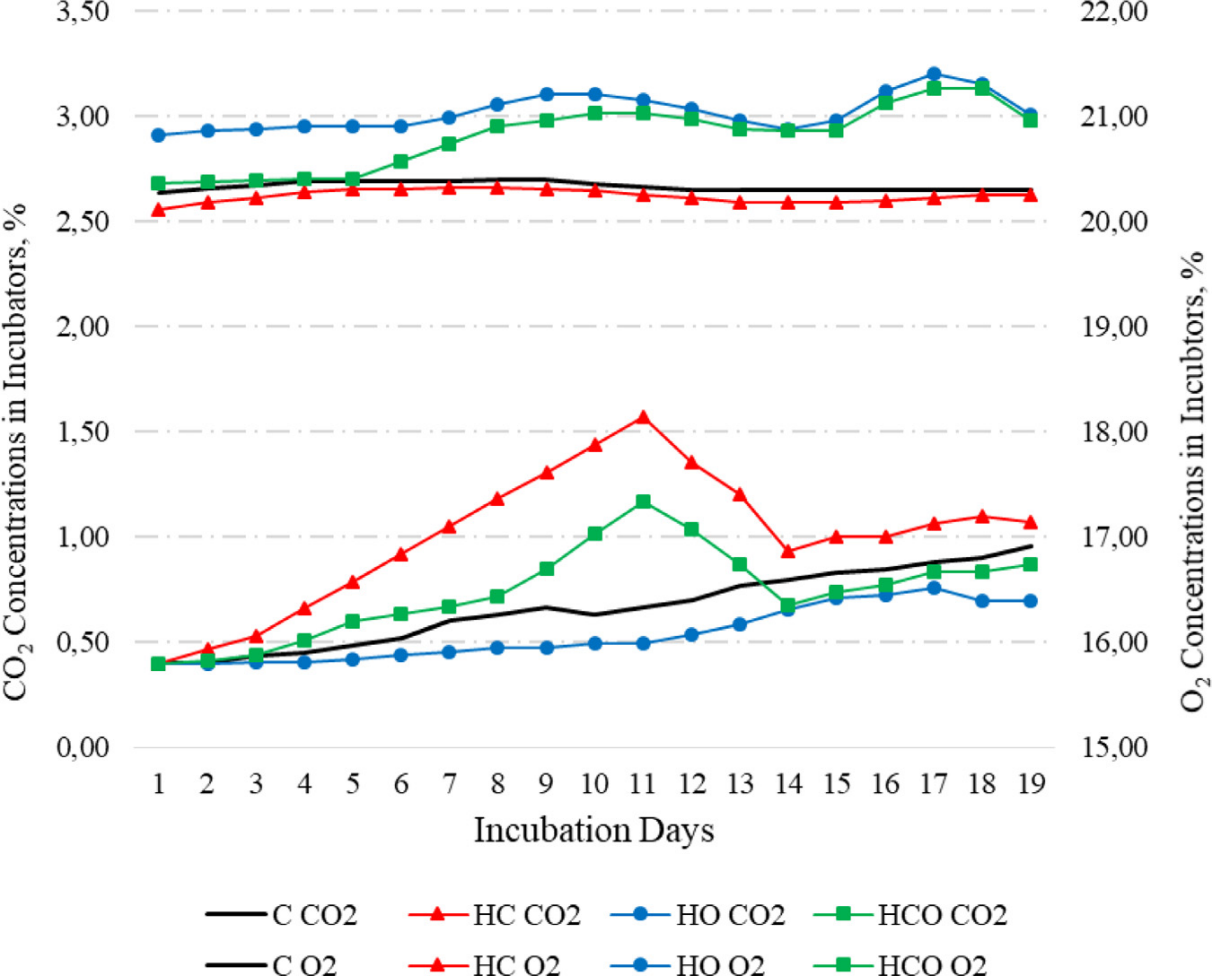
The measured CO_2_ and O_2_ concentrations in the diffrent incubator ventilation programs during the experiment. Incubator ventilation program, C: Control (0.67% CO_2_ and 20.33% O_2_), HC: High CO_2_ (1.57% CO_2_ and 20.26% O_2_), HO: High O_2_ (0.50% CO_2_ and 21.16% O_2_), HCO: High CO_2_ + O_2_ (1.17% CO_2_ 21.03% O_2_).

Given the results obtained from the IVP groups, the CO_2_ concentrations were found to have gradually increased during the first 10 d in the HC (high CO_2_) and HCO (high CO_2_ + high O_2_) groups. Similarly, the CO_2_ concentrations in the incubators were found to be 0.50, 0.67, 1.17, and 1.57% in the HO, C, HCO and HC groups, respectively, on the 10th d of incubation. In parallel with the increase in the CO_2_ concentrations, the O_2_ concentrations in the incubators gradually decreased to 21.16, 20.33, 21.03, and 20.26% in the HO, C, HCO, and HC groups, respectively. Finally, the averages of CO_2_–O_2_ percentages of the IVP treatments during the experiment were 0.67 to 20.33 O_2_ for C, 1.03 to 20.26 for HCO, 0.50 to 21.00 for HO and 0.79 to 20.81 for HCO groups (Figure 1).

The F values of the IVP groups (C, HC, HO, HCO) were 89.64, 90.27, 86.69, and 86.65%, respectively. The numerical differences were between the F values of the groups, and these differences were lower between the C and HC groups than the HO and HCO groups. However, the differences were not significant (*P* > 0.05). It was suggested that the insignificant differences between F values of the IVP groups were normal and that flock uniformity, trial design and egg lay-out plan were good, thus the effect of EW was eliminated. Also, the differences between the F values of PSA × IVP interaction groups were significant (*P* < 0.05) due to the F value of O group being lower than the other PSA groups.

Contrary to F values, numerical differences in the EED and MLPED values of the IVP groups were found and some of these differences were evident. The highest EED values were found in the HC group and the highest MLPED values were found in the C group. Therefore, the differences between the EED value of the HC group and the C and HCO groups were found to be significant (*P* < 0.05). Like EED values of the IVP groups, it was found that the differences between the MLPED value of the C group and, HO and HCO groups were statistically significant (*P* < 0.05).

As with the EED and MLPED values, numeric differences were found in the HFE values of the IVP treatment groups, where the lowest HFE was found in the C group (*P* < 0.05).

Furthermore, the PSA × IVP interaction has effects on EED, MLPED, and HFE (*P* < 0.05). The lowest EED values were obtained from YC, YHO, MC, MHC, MHO, MHCO, OC, OHO, and OHCO groups, conversely the highest EED values were found in the OHC group for PSA × IVP interaction and the differences between groups were found to be significant (*P* < 0.05). Like EED values, highest MLPED values in the YC and OC groups, lowest MLPED values in the YHO, YHCO, MHC, MHO, MHCO, OHO, and OHCO groups were found. The evident numerical differences between the MLPED values of these groups were also statistically significant for PSA × IVP interaction (*P* < 0.05).

The data regarding the incubation performance showed that IVP affected EED, MLPED, and consequently HFE. The results concerning EED, MLPED, and HFE were in line with many previous studies (Decuypere et al., 2001, 2006; De Smit et al., 2006; Everaert et al., 2007), but not with several (Onagbesan et al., 2007; Piestun et al., 2008).

The significant differences between the EED in the HC treatment groups and the others especially in the O of PSA groups indicated that greater CO_2_ values (0.50–1.57%) could be harmful during incubation. Similarly, significant differences between MLPED and HFE in the O groups and the C groups especially in the Y and O of PSA groups indicated that greater O_2_ values (20.26–21.16%) could be beneficial. The results of higher rates of EEDs, MLPED and HFE in the HO group were not consistent with those of other researchers (Taylor et al., 1971), who reported increased O_2,_ and CO_2_ were compensated over time. In this respect, the current results do not support those found in the literature (Ar et al., 1974). This can be attributed to the change in eggshell conductance depending on EW and enhancement in machinery in the last decades.

High MLPED and low HFE rates in the control groups are considered to have been affected by both CO_2_ and O_2_ treatments.

When the HFE values of the group for PSA × IVP interaction were evaluated, prominently lower HFE value was found in the OC group than the YHO, YHCO, MC, MHC, MHO, MHCO, and OHCO groups, and the differences were statistically significant (*P* < 0.05).

This is considered to be as result of the change in eggshell conductance and pore structure due to the gaseous composition and barometric pressure of ambient fresh air, and possible negative effects were physiologically refrained at these concentrations. However, the main aim of this study was to investigate whether these differences in O_2_ concentration, corresponding to approximately 10%, had an improving impact on embryonic deaths, and consequently on HFE which would have been resulted by positive effects of O_2_ supplementation for these conditions. As mentioned, many private companies operate their hatcheries and broiler chick breeding farms under these conditions, and therefore, the experiment was important to present the causes of performance loss related to the CO_2_ and O_2_ concentrations.

Similar to our findings, some researchers reported that increasing the CO_2_ concentrations from 1.00 to 1.50% gradually during first 10 days of incubation resulted in improved embryonic growth, encouraged early hatching and increased H in turkey and chicken eggs (Tona et al., 2007). As suggested by Visschedijk (1991), this may be due to the functional conductance of eggshell along with gaseous composition and barometric pressure of the ambient fresh air.

Considering altitude of the experiment laboratory (822 m), the findings support the reports of breeder companies suggesting that hatcheries should be established in areas at a maximum altitude of 750 m (Cobb, 2013) or 1,500 m (Tullett, 2013) in order to obtain better HFE values. This is a result of the presence of lower O_2_ concentrations during the incubation period at lower altitudes, as also confirmed by the results of the present study. Therefore, for areas at a similar and higher altitudes of the study area in this study (822 m), the use of an O_2_ concentrator may be useful considering that the amount of changes in the CO_2_ and O_2_ concentrations especially in the first 10 days of incubation (0.50–1.57% and 20.26–21.16%, respectively) did not have any effect on embryonic deaths in the present study.

However, more detailed studies on this subject, considering the air pressure with partial pressures of gases and including the growing and even slaughtering stages, will help us to better understand the relevant physiological processes related to the possible problems that may be seen in the field and to eliminate the hesitations.

### Hematologic Analysis

Red blood cell count, PCV, and Hb values of the hatched chicks of the C, HO, HC, and HCO groups in young, middle-age, and old broiler parental flocks are given in Table 3.

**Table 3:** Effects of different O_2_/CO_2_ ventilation programs on RBC, PCV, and Hb values in newly hatched chicks from different parental stock ages (M ± SEM).

PSA	IVP	Treatment groups	RBC × 10^3^/mm^3^	PCV %	Hb g/dL
Y	C	YC	18.79 ± 0.84	28.00 ± 1.14	6.52 ±1.72
	HC	YHC	19.98 ± 1.03	29.17 ± 0.91	7.01 ± 1.95
	HO	YHO	17.64 ± 2.79	27.88 ± 2.72	9.48 ± 0.52
	HCO	YHCO	15.86 ± 0.73	27.38 ± 1.13	9.01 ± 0.39
M	C	MC	17.56 ± 0.75	29.83 ± 1.64	7.47 ± 1.30
	HC	MHC	19.29 ± 2.10	31.57 ± 1.91	10.01 ± 1.56
	HO	MHO	17.38 ± 1.61	28.63 ± 1.76	9.38 ± 0.71
	HCO	MHCO	20.86 ± 1.30	29.13 ± 0.61	9.89 ± 0.40
O	C	OC	16.89 ± 1.09	28.14 ± 1.03	7.25 ± 1.25
	HC	OHC	19.40 ± 1.73	28.63 ± 1.00	9.77 ± 0.51
	HO	OHO	15.61 ± 1.52	27.00 ± 1.23	9.37 ± 0.55
	HCO	OHCO	15.94 ± 2.40	30.63 ± 2.05	10.90 ± 0.83

Abbreviations: PSA, parental stock age, Y, young (29 wk), M, middle (37 wk), O, old (55 wk); IVP, incubator ventilation program, C, control (0.67% CO_2_ and 20.33% O_2_), HC, high CO_2_ (1.57% CO_2_ and 20.26% O_2_), HO, high O2 (0.50% CO_2_ and 21.16% O_2_), HCO, high CO_2_ + O_2_ (1.17% CO_2_ 21.03% O_2_); RBC, red blood cell, PCV, packed cell volüme, Hb, hemoglobin.

As shown in table, there were no changes in the groups which were exposed to different IVP.

Neither RBC count nor PCV and Hb values were affected by high O_2_ (HO) and CO_2_ (HC) or both (HCO). The results of the present study are similar to those of Maxwell et al. (1987) who reported no changes in hematologic parameters in the O_2_ supplemented group and Beker et al. (1995) who indicated that there were no differences in erythrocyte, leukocyte, hematocrit, and hemoglobin values in low O_2_ (or HC) concentrations. Furthermore, Tong et al. (2015) reported that there was no effect of hypercapnia on PCV and Hb values.

### Hormone Analysis

While the C group was compared with the treatment groups, HC group was compared with the HCO group.

**Figure 2:**
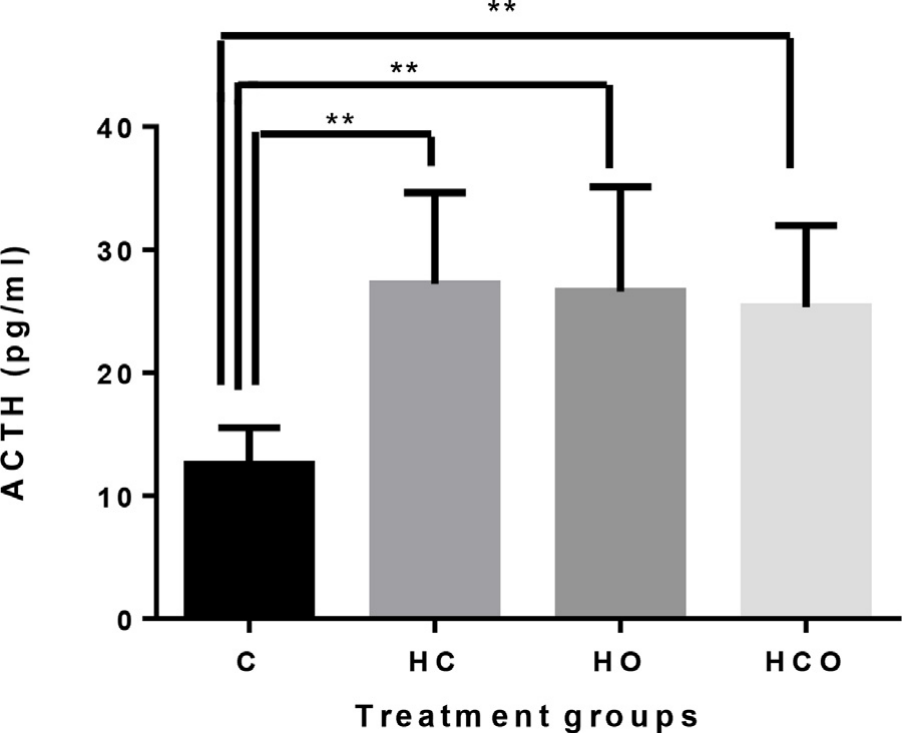
Plasma ACTH concentrations in hatched chicks from middle-aged (37 wk) parental stocks exposed to different incubator ventilation programs. ** Shows the difference among the groups (*P* < 0.01). Incubator ventilation program, C: Control (0.67% CO_2_ and 20.33% O_2_), HC: High CO_2_ (1.57% CO_2_ and 20.26% O_2_), HO: High O_2_ (0.50% CO_2_ and 21.16% O_2_), HCO: High CO_2_ + O_2_ (1.17% CO_2_ 21.03% O_2_).

As shown in Figure 2, the plasma ACTH concentration in all treatment groups were found to be higher than the C group (*P* < 0.01). IVP also increased ACTH level by causing stress in the middle parental stock age, however this increase did not affect the fertility and hatchability rate.

**Figure 3:**
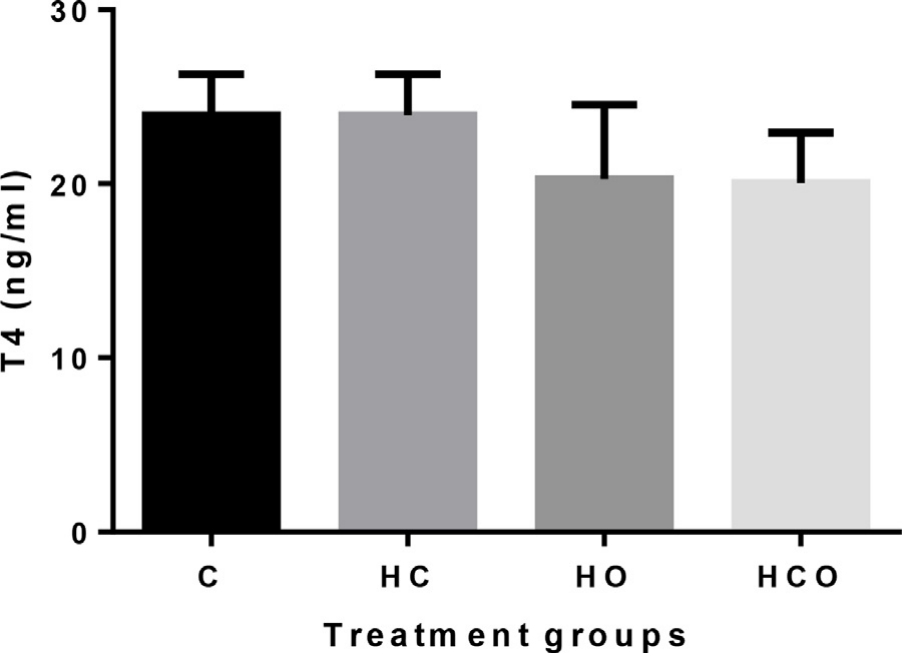
Plasma T_4_ concentrations in hatched chicks from middle-aged (37 wk) parental stocks exposed to different incubator ventilation programs. Incubator ventilation program, C: Control (0.67% CO_2_ and 20.33% O_2_), HC: High CO_2_ (1.57% CO_2_ and 20.26% O_2_), HO: High O_2_ (0.50% CO_2_ and 21.16% O_2_), HCO: High CO_2_ + O_2_ (1.17% CO_2_ 21.03% O_2_).

There was no difference in T_4_ concentration among the control group and the treatment groups, or among the treatment groups (*P* > 0.05; Figure 3).

**Figure 4:**
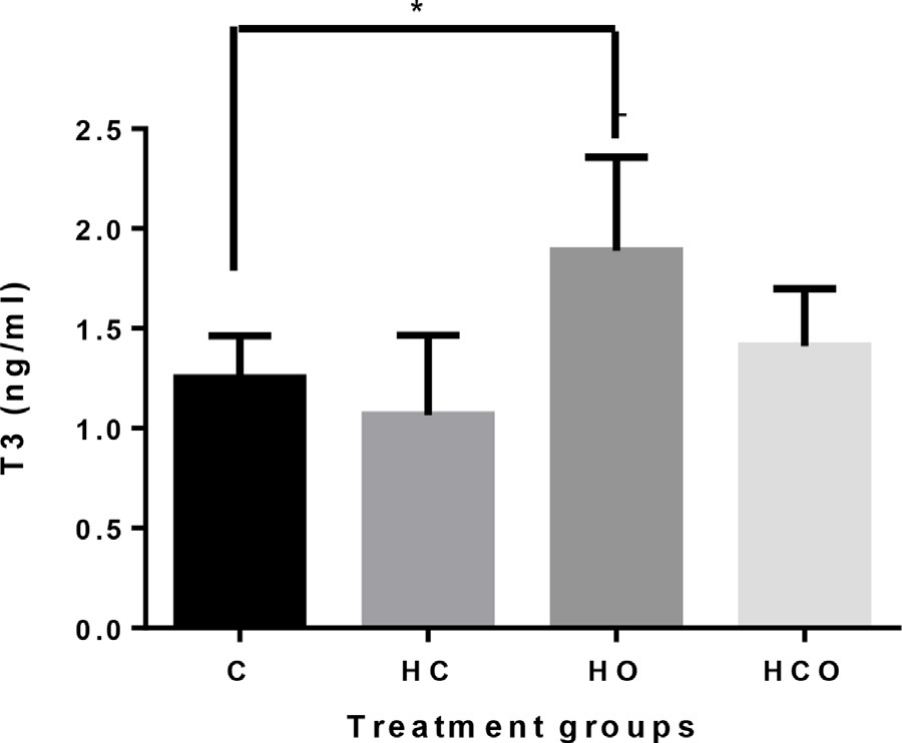
Plasma T_3_ concentrations in hatched chicks from middle-aged (37 wk) parental stocks exposed to different incubator ventilation programs. *Shows the difference among the groups (*P* < 0.05). Incubator ventilation program, C: Control (0.67% CO_2_ and 20.33% O_2_), HC: High CO_2_ (1.57% CO_2_ and 20.26% O_2_), HO: High O_2_ (0.50% CO_2_ and 21.16% O_2_), HCO: High CO_2_ + O_2_ (1.17% CO_2_ 21.03% O_2_).

The plasma T_3_ concentration in the HO group was higher than the control group and HC group (*P* < 0.05; Figure 4).

O_2_ and CO_2_ exchange is vital for the embryonic cells. If the eggs are stored in a closed environment, gas exchange related problems affect egg quality, H, pipping, and the development of the embryo (Decupeyre et al., 2001). In modern hatcheries, chicken eggs are incubated in the presence of 21% O_2_. Due to the increased metabolic activities in the second half of the incubation, CO_2_ rate increases and the growing embryo need more O_2_ (Stock and Metcalfe, 1987). Incubators are designed to provide O_2_ to the embryos and exhaust excessive CO_2_ from the machine (Onagbesan et al., 2007). The quality of the O_2_ varies with altitude. It decreases with high altitude which affects the incubation period and H (Visschedjik, 1991; Hassanzadeh et al., 2004).

Corticosterone, which is released by ACTH, is involved in the maintenance of the homeostasis by metabolism and stress regulation (Scott et al., 1981). Therefore, its concentration in embryos can influence the postnatal life of the chick (Meeuwis et al., 1989). Hypoxia or hypercapnia, or both during the first half of incubation stimulate blood vessel development and enhance embryo growth, stimulate early hatching and increase the H rate. At the end of incubation, hypoxia, or hypercapnia may also regulate pipping and hatching events and cardiovascular or pulmonary changes. Decupeyre et al. (2006) and De Smith et al. (2006) indicated that hypoxic conditions at the end of the incubation period increased corticosterone concentrations and hatching rate. In this study, the increment of the ACTH concentration in the HO, HC, and HCO groups compared to the C group may be related with the added O_2_ and CO_2_ that may cause hyperoxic and hypoxic related stress-induced ACTH stimulation. Also, our result was consistent with Hassanzadeh et al. (2004) who reported that corticosterone was increased in high altitude in chick embryos. Although treatment groups have high ACTH levels, F and H rate did not change by these O_2_/CO_2_ levels.

Triiodothyronine, thyroxine, and corticosterone concentrations can change depending on the O_2_ and CO_2_ concentrations in the incubator at higher altitudes. Different CO_2_ concentrations alter the T_3_, T_4_, and corticosterone concentrations and the differences have not been found to be significant except for the corticosterone concentrations of hatched chicks in middle-aged broiler parental flocks (De Smit et al., 2006).

As T_3_, T_4_, and corticosterone hormones play a role in pipping and hatching, chick embryos need them during incubation which may affect their livability (Tullett, 2009). In the HO group in this study, the main air inlet was always open during the 21 d and only O_2_ was increased to 10% using an O_2_ concentrator. The HO group was exposed to the highest O_2_ during the incubation period. Increased T_3_ concentration in the HO group may have been caused by the high O_2_ concentrations. Contrary to the results of the present study, Sahan et al. (2011) reported that O_2_ supplementation during the incubation period did not change the T_3_ and T_4_ concentrations at high altitude (1,100 m). In addition, Bahadoran et al. (2010) reported the same results in incubated chicks at 1,800 m above sea levels. The reason why T_3_ concentration increased in the HO group in the present study might be due to altitude difference. The altitude of the study area in the present study was not as high as the aforementioned studies. It is thought that the amount of given O_2_ may have increased the T_3_ concentration by stimulating the metabolism resulting in a lower drop in O_2_ concentration in the present study. Probably, due to the conversion of T_4_ to T_3_, T_4_ concentrations were not found to be different between the treatment groups.

### Histopathological Findings

**Figure 5:**
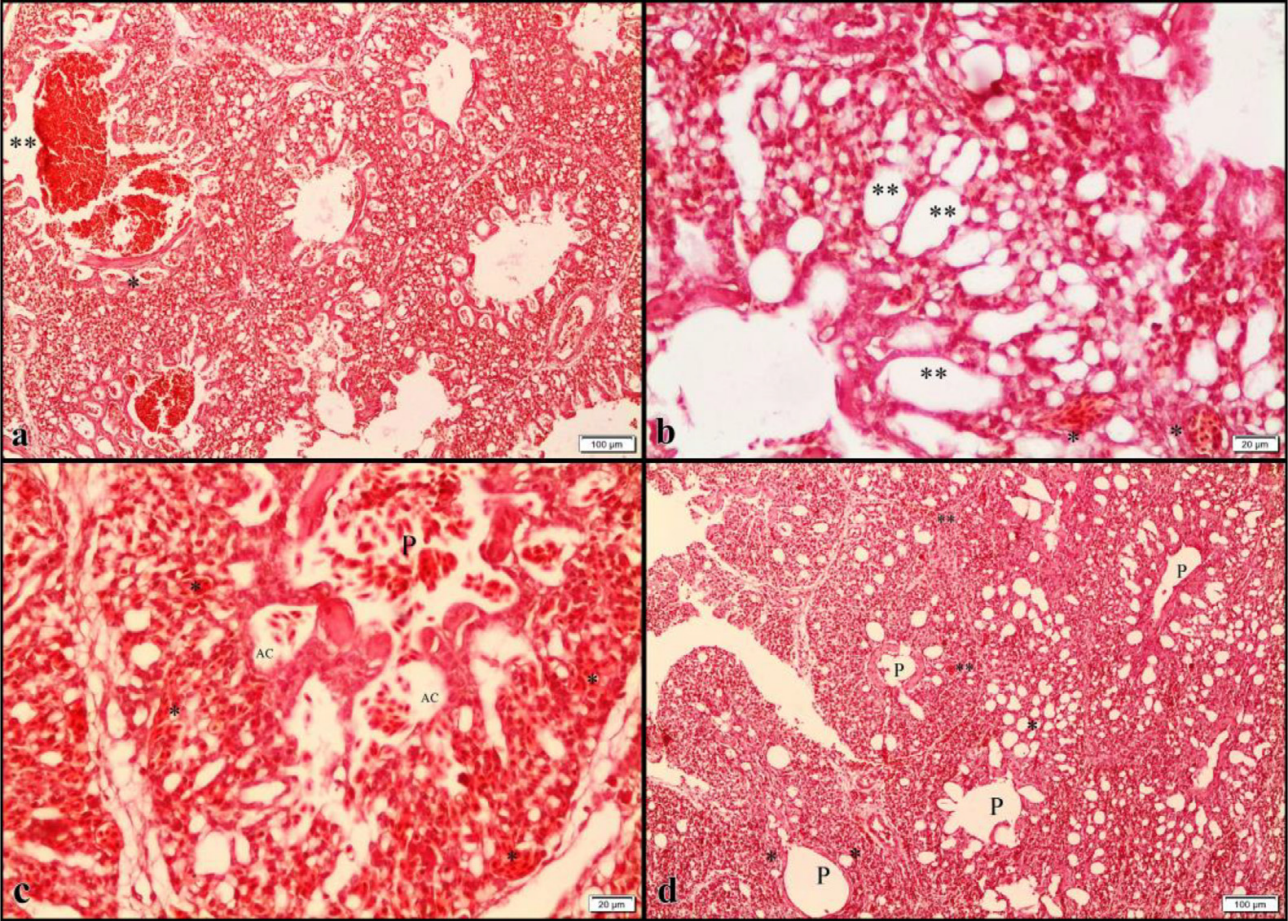
Histopathological findings detected in the lungs^#^. ^#^Parental stock Age, Y (young, 29 wk), M (middle-aged, 37 wk, O (old, 55 wk.). Incubator ventilation program, C: Control (0.67% CO_2_ and 20.33% O_2_), HC: High CO_2_ (1.57% CO_2_ and 20.26% O_2_), HO: High O_2_ (0.50% CO2 and 21.16% O_2_), HCO: High CO_2_ + O_2_ (1.17% CO_2_ 21.03% O_2_). ^a^YHO lung; peribronchial hemorrhage (**) and hyperemia in air capillaries (*), Bar: 100 μm, Hematoxylin and Eosin. ^b^MHCO lung; hyperemia (*), emphysema in air capillaries (**), Bar: 20 μm, Hematoxylin and Eosin. ^c^OHC lung; peribronchial hemorrhage (P) with severe hyperemia in all vessels (*), emphysema in air capillaries (AC), and hemorrhage (P), Bar: 100 μm, Hematoxylin and Eosin. ^d^MC lung; very mild hyperemia (**), mild emphysema in air capillaries (*) and peribronchials in normal view (P), Bar: 100 μm, Hematoxylin and Eosin.

**Figure 6:**
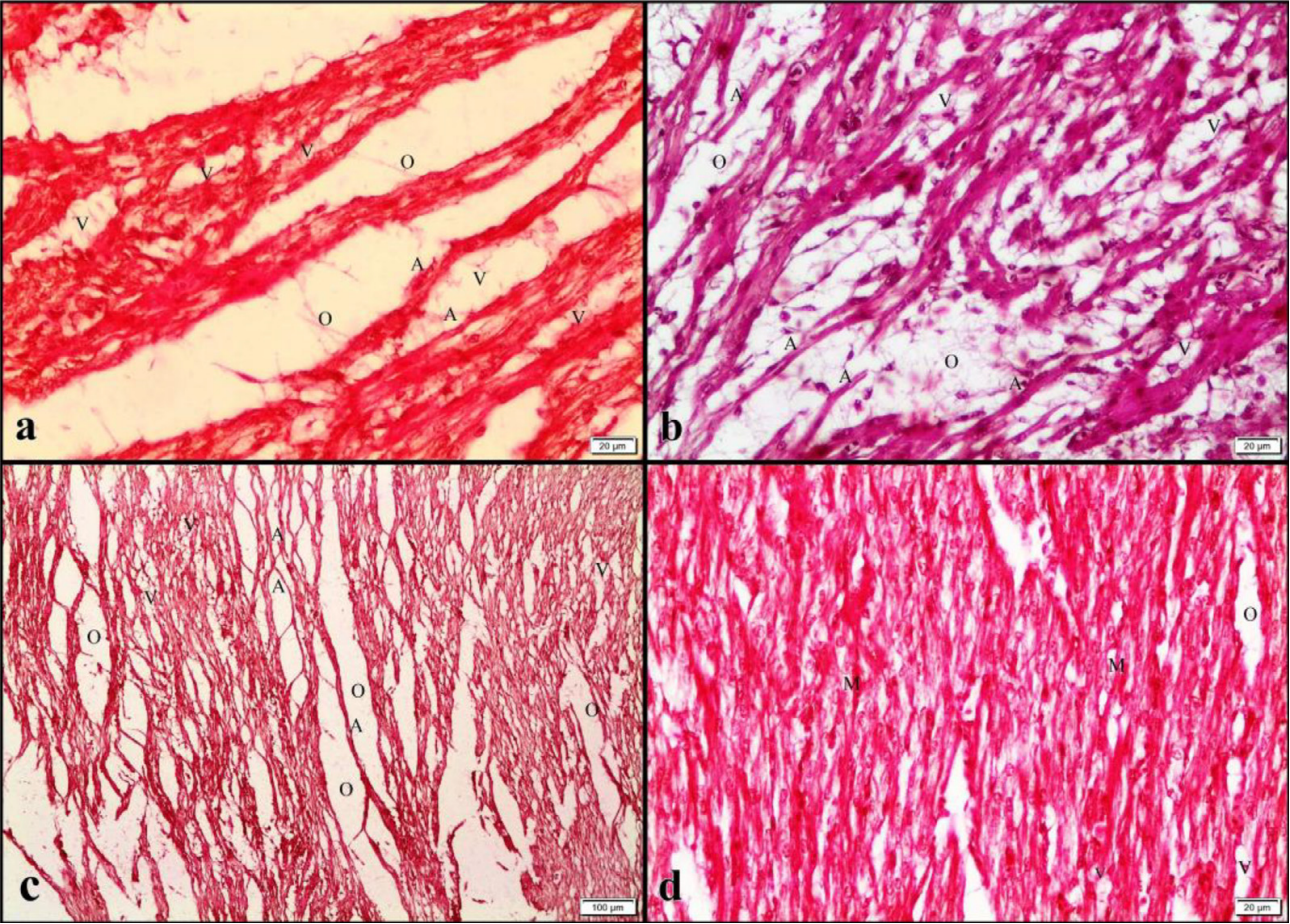
Histopathological findings detected in the hearts^#^. ^#^Parental stock age, Y (young, 29 wk), M (middle-aged, 37 wk, O (old, 55 wk). Incubator ventilation program, C: Control (0.67% CO_2_ and 20.33% O2), HC: High CO_2_ (1.57% CO_2_ and 20.26% O_2_), HO: High O_2_ (0.50% CO_2_ and 21.16% O_2_), HCO: High CO_2_ + O_2_ (1.17% C_O2_ 21.03% O_2_). ^a^YHC heart; severe vacuolization (V), edema (O), and atrophy (A) in myofibrils, Bar: 20 μm, Hematoxylin and Eosin. ^b^MHCO heart; Vacuolization (V), edema (O), and atrophy (A) in myofibrils, Bar: 20 μm, Hematoxylin and Eosin. ^c^Group OHC heart; severe vacuolization (V), edema (O), and atrophy (A) in myofibrils, Bar: 100μm, Hematoxylin and Eosin. ^d^OC heart; in some of the relatively normal myofibrillations, mild vacuolization (V), and edema (O), Bar: 20 μm, Hematoxylin and Eosin.

In the histopathological examination, hyperemia, hemorrhage with emphysema in the air capillaries and peribronchial hemorrhage was observed at different intensities in the lung tissue in all age groups except for the control groups (Figure 5). Similarly, histopathological examination of the heart revealed vacuolation, edema, and atrophy in myofibrils (Figure 6). The histopathological findings and the severity of the groups are summarized in Tables 4 and 5.

**Table 4:** Histopathological findings detected in the lungs.

PSA	IVP	Treatment Groups	Parabronchial hemorrhage	Emphysema in air capillaries	Hemorrhage in air capillaries	Hyperemia
Y	C	YC	-	-	-	+
	HC	YHC	++	+	++	+
	HO	YHO	+++	+	++	++
	HCO	YHCO	++	+	++	++
M	C	MC	-	-	±	±
	HC	MHC	++	++	++	++
	HO	MHO	+	+	++	+
	HCO	MHCO	±	+++	±	++
O	C	OC	-	-	-	-
	HC	OHC	+++	++	+++	+++
	HO	OHO	++	+++	++	++
	HCO	OHCO	+++	++	+++	+++

Abbreviations: PSA, parental stock age, Y, young (29 wk), M, middle (37 wk), O, old (55 wk); IVP, incubator ventilation program, C, control (0.67% CO_2_ and 20.33% O_2_), HC, high CO_2_ (1.57% CO_2_ and 20.26% O_2_), HO, high O_2_ (0.50% CO_2_ and 21.16% O_2_), HCO, high CO_2_ + O_2_ (1.17% CO_2_ 21.03% O_2_).

-: none, ±: very light, +: light, ++: moderate, +++: severe.

**Table 5:** Histopathological findings detected in the hearts.

PSA	IVP	Treatment groups	Edema	Vacuolation of myofibers	Atrophy
Y	C	YC	-	±	-
	HC	YHC	+++	+++	+++
	HO	YHO	+++	+++	+++
	HCO	YHCO	++	++	++
M	C	MC	±	±	±
	HC	MHC	++	++	++
	HO	MHO	+++	+++	+++
	HCO	MHCO	++	+	++
O	C	OC	±	±	±
	HC	OHC	+++	+++	+++
	HO	OHO	+++	+++	+++
	HCO	OHCO	+++	+++	+++

Abbreviations: PSA, parental stock age, Y, young (29 wk), M, middle (37wk), O, old (55 wk); IVP, incubator ventilation program, C, control (0.67% CO_2_ and 20.33% O_2_), HC, high CO_2_ (1.57% CO_2_ and 20.26% O_2_), HO, high O_2_ (0.50% CO_2_ and 21.16% O_2_), HCO, high CO2 + O2 (1.17% CO_2_ 21.03% O_2_).

-: none, ±: very light, +: light, ++: moderate, +++: severe.

Chronic and acute hypercapnia, hyperoxia or hypoxia have been reported to influence the development (morphological and physiological) of chick embryo and their effects may depend on the timing of their application during incubation (Altimiras and Phu, 2000; Mortola, 2004; Chan and Burggren, 2005; Onagbesan et al., 2007). The tolerance of embryos to hyperoxia increases further between the 13th and 16th days of incubation (Onagbesan et al., 2007). Between the 16th and 18th d, the tolerance of the embryo to hyperoxia shifted again to a lower concentration (Stock et al., 1983; Stock and Metcalfe 1987; Onagbesan et al., 2007). Haring et al. (1970) reported that the high embryonic mortality seen after the exposure to high concentration of CO_2_ (6 %) for 24 h at any time during the first 10 days of incubation resulted from noncardiac and cardiac malformations. In the light of this literature, it was thought that the lesions (observed in HO, HCO, and HC groups) both in the heart and lung were formed as a result of the hypercapnia and hyperoxia.

In the literature review, it was concluded that these lesions may be formed due to changes in the acid-base balance. Responses in acid–base balance to 1-d exposure to altered environmental gas mixtures differ depending on the gas mixture and age of chicken embryos (Burggren et al., 2012). One day of hypercapnic exposure (5% CO_2_, 20% O_2_) increases PaCO_2_ and decreases pHa, producing respiratory acidosis that is partially compensated by metabolic alkalosis at all embryonic stages examined. Similar patterns of partially compensated respiratory acidosis have been reported in embryos exposed to 9% CO2 in air for >3 d (Dawes and Simkiss, 1969). One day of exposure to hypercapnic hypoxia (5% CO_2_, 15% O_2_) abolishes compensatory metabolic alkalosis in d 15 and d 17 embryos, but a metabolic compensation of ∼37% still occurs in d 13 embryos (Mueller et al., 2015).

Therefore, it has been observed that the use of an oxygen concentrator to increase the reduced oxygen level in the altitude and higher altitudes of the study or keeping the carbon dioxide level high in the first ten days of incubation has different effects. Although these applications have some positive effects on the incubation results with the effect of PSA, it has been determined that they have negative effects such as hyperoxia or hypoxia on tissue development and some blood values.

The overall results of the experiment revealed that increasing the CO_2_ and O_2_ to certain levels in incubator can provide improvement in embryonic deaths and hatchability of fertile eggs, while hypoxic/hypercapnic or hyperoxic conditions caused stress on the birds and their ACTH levels increased in all treatment groups. In addition, vacuolization and hemorrhage in the lungs and heart of all PSA groups except for the control group were determined. Therefore, keeping carbon dioxide levels high during the first ten days of incubation or using an O_2_ concentrator seems to be unnecessary at such altitudes and higher, however, can be evaluated in places higher than the altitude of 822 m by data to be obtained from future studies considering embryonic development and the field performance of chicks.

## Appendix Supplementary materials

Image, application 1

## References

[bib0001] Abiola S.S., Meshioye O.O., Oyerinde B.O., Bamgbose M.A. (2008). Effect of egg size on hatchability of broiler chicks. Arch. Zootec..

[bib0002] Ahmed M., Biswas A., Roy B.G., Srivastava R.B. (2013). Frequently encountered problems during hatching in cold arid high altitude regions such as Ladakh in India: causes and remedies. Worlds Poult. Sci. J..

[bib0003] Altan O., Sahan U., Ipek A., Aydin C., Bayraktar H. (2006). Effects of oxygen supplementation on embryonic survival, haematological parameters and plasma glucose level of broiler chicks. Arch. Geflügelk..

[bib0004] Altimiras J., Phu L. (2000). Lack of physiological plasticity in the early chicken embryo exposed to acute hypoxia. J. Exp. Zool..

[bib0005] Ar A., Deeming D.C. (2009). Water and gas exchange in determining hatchability success. Avian Biol. Res..

[bib0006] Ar A., Paganelli C.V., Reeves R.B., Greene D.G., Rahn H. (1974). The avian egg: water vapour conductance, shell thickness and functional pore area. Condor.

[bib0008] Aviagen (2016).

[bib0009] Bahadoran S., Hassanzadeh M., Zamanimoghaddam A. (2010). Effect of chronic hypoxia during the early stage of incubation on prenatal and postnatal parameters related to ascites syndrome in broiler chickens. Iran J. Vet. Res..

[bib0010] Beker A., Vanhoser S.L., Teeter R.G. (1995). Pages 285-291 in Avian Disease.

[bib0011] Blacker H.A., Orgeig S., Daniels C.B. (2004). Hypoxic control of the development of the surfactant system in the chicken: evidence for physiological heterokairy. Am. J. Physiol. Regul. Integr. Comp. Physiol..

[bib0012] Burggren W.W., Andrewartha S.J., Tazawa H. (2012). Interactions of acid–base balance and hematocrit regulation during environmental respiratory gas challenges in developing chicken embryos (Gallus gallus). Resp. Physio. Neurob..

[bib0013] Campbell T.W., Ellis C.K. (2007).

[bib0014] Chan T., Burggren W. (2005). Hypoxic incubation creates differential morphological effects during specific developmental critical windows in the embryo of the chicken (*Gallus gallus*). Respir. Physiol. Neurobiol..

[bib0015] Cobb (2013).

[bib0016] Currie R.J.W. (1999). Ascites in poultry: recent investigations. Avian Pathol.

[bib0017] Dawes C., Simkiss K. (1969). The acid-base status of the blood of the developing chick embryo. J. Exp. Biol.

[bib0018] De Smit L., Bruggeman V., Debonne M., Tona J.K., Kamers B., Everaert N., Witters A. (2008). The effect of nonventilation during early incubation on the embryonic development of chicks of two commercial broiler strains differing in ascites susceptibility. Poult. Sci..

[bib0019] De Smit L., Bruggeman V., Tona J.K., Debonne M., Onagbesan O., Arckens L., De Baerdemaeker J., Decuypere E. (2006). Embryonic developmental plasticity of the chick: increased co_2_ during early stages of incubation changes the developmental trajectories during prenatal and postnatal growth. Comp. Biochem. .Physiol. Part A..

[bib0020] Decuypere E., Onagbesan O., De Smit L., Tona K., Everaert N., Witters A., Debonne M., Verhoelst E., Buyse J., Hassanzadeh M., De Baerdemaeker J., Arckens L., Bruggeman V. (2006). EPC 2006 - 12th European Poultry Conference.

[bib0021] Decuypere E., Tona K., Bruggeman V., Bamelis F. (2001). The day-old chick: a crucial hinge between breeders and broilers. Worlds Poult. Sci. J..

[bib0022] Deeming C.D., Ferguson M.W. (1991). Egg Incubation: Its Effects on Embryonic Development in Birds and Reptiles.

[bib0023] Elibol O., Brake J. (2008). Effect of egg weight and position relative to incubator fan on broiler hatchability and chick quality. Poult. Sci..

[bib0024] Everaert N., Kamers B., Witters A., De Smit L., Debonne M., Decuypere E., Bruggeman V. (2007). Effect of four percent carbon dioxide during the second half of incubation on embryonic development, hatching parameters and posthatch growth. Poult. Sci..

[bib0025] Fernandes J., Bortoluzzi C., Esser A.F.G., Contini J.P., Stokler P.B., Faust D. (2014). Performance of broilers submitted to high co_2_ levels during incubation combined with temperature fluctuations at late post-hatch. Braz. J. Poult. Sci..

[bib0026] Haring O.H., Patterson J.R., Sarch M.A. (1970). Prenatal development of the cardiovascular system in the chicken. Arch. Pathol..

[bib0027] Hassanzadeh M., Bozorgmehri F., Buyse J., Bruggeman V., Decuypere E. (2004). Effect of chronic hypoxia during embryonic development on physiological functioning and on hatching and post-hatching parameters related to ascites syndrome in broiler chickens. Avian Pathol.

[bib0028] Hintze J.L. (2011).

[bib0029] Huwaida E.E., Sakin M.A.I.Y., Elagib H.A.A., Bakhiet E., Dousa B.M., Elamin K.M. (2015). Effect of egg weight and egg shell thickness on hatchability and embryonic mortality of Cobb broiler breeder eggs. Global J. Anim. Sci. Res..

[bib0030] Jozsa R., Vigh S., Mess B., Schally A. (1986). Ontogenic development of corticotropin-releasing factor (CRF)-containing neural elements in the brain of the chicken during incubation and after hatching. Cell Tissue Res.

[bib0031] Julian R.J. (2000). Physiological, management and environmental triggers of the ascites syndrome: a review. Avian Pathol.

[bib0032] Kocabas Z., Ozkan M., Baspinar E. (2013).

[bib0033] Lourens A., Van Den Brand H., Hetkamp M.J.W., Meijerhof R., Kemp B. (2007). Effects of egg shell temperature and oxygen concentration on embryo growth and metabolism during incubation. Poult. Sci..

[bib0034] Maatjens C.M., Reijrink A.M., Molenaar R., Van Der Pol C.W., Kemp B., Van Den Brand H. (2014). Temperature and CO_2_ during the hatching phase. I. Effects on chick quality and organ development. Poult. Sci..

[bib0035] Maatjens C.M., Reijrink A.M., Molenaar R., Van Der Pol C.W., Kemp B., Van Den Brand H. (2014). Temperature and CO_2_ during the hatching phase. II. Effects on chicken embryo physiology. Poult. Sci..

[bib0036] Maxwell M.H., Robertson G.W., Moseley D. (1995). Serum troponin t values in 7-day-old hypoxia and hyperoxia-treated, and 10-day-old ascitic and debilitated, commercial broiler chicks. Avian Pathol.

[bib0037] Maxwell M.H., Tullett S.G., Burton F.G. (1987). Haemotology and morphological changes in young broiler chicks with experimentally induced hypoxia. Res. Vet. Sci..

[bib0038] Meeuwis R., Michielsen R., Decuypere E., Kuhn E.R. (1989). Thyrotropic activity of the ovine corticotropin-releasing factor in the chick embryo. Gen. Comp. Endocrinol..

[bib0039] Meijerhof R. (2009). The influence of incubation on chick quality and broiler performance. Proc. Aust. Poult. Sci. Symp..

[bib0040] Mersten-Katz C., Barnea A., Yom-Tov Y., Ar A. (2013). The woodpecker's cavity microenvironment: advantageous or restricting?. Avian Biol. Res..

[bib0041] Molenaar R., Meijerhof R., Van Den Anker I., Hetkamp M.J.W., Van Den Borne J.J.G.C., Kemp B., Van Den Brand H. (2010). Effect of eggshell temperature and oxygen concentration on survival rate and nutrient utilization in chicken embryos. Poult. Sci..

[bib0042] Mortola J.P. (2004). Ventilatory response to hypoxia in the chick embryo. Comp. Biochem. Physiol. A: Integ. Physiol..

[bib0043] Mueller C.A., Burggren W.W., Tazawa H. (2015). Pages 739-766 in Sturkie's Avian Physiology.

[bib0044] Onagbesan O., Bruggeman V., De Smit L., Debonne M., Witters A., Tona K., Everaert N., Decuypere E. (2007). Gas exchange during storage and incubation of avian eggs: effects on embryogenesis, hatchability, chick quality and post-hatch growth. Worlds Poult. Sci. J..

[bib0045] Ozlu S., Ucar A., Banwell R., Elibol O. (2019). The effect of increased concentration of carbon dioxide during the first 3 days of incubation on albumen characteristics, embryonic mortality and hatchability of broiler hatching eggs. Poult. Sci..

[bib0046] Piestun Y., Shinder D., Ruzal M., Halevy O., Brake J., Yahav S. (2008). Thermal manipulations during broiler embryogenesis: effect on the acquisition of thermotolerance. Poult. Sci..

[bib0047] Sahan U., Ipek A., Yilmaz-Dikmen B., Aydin C., Kederli E. (2011). Effect of oxygen supplementation in the hatcher at high altitude on the incubation results of broiler eggs laid at low altitude. Brit. Poult. Sci..

[bib0048] Santos F.S.D.L., Tellez G., Farnell M.B., Balog J.M., Anthony N.B., Pavlidis H.O., Donoghue A.M. (2005). Hypobaric hypoxia in ascites resistant and susceptible broiler genetic lines influences gut morphology. Poult. Sci..

[bib0049] Scott T.R., Johnson W.A., Satterlee D.G., Gildersleeve R.P. (1981). Circulating levels of corticosterone in these rum of developing chick embryos and in newly hatched chick. Poult. Sci..

[bib0050] SPSS (2013).

[bib0051] Stock M.K., Francisco D.L., Metcalfe J. (1983). Organ growth in chick embryos incubated in 40% or 70% oxygen. Respir. Physiol..

[bib0052] Stock M.K., Metcalfe J. (1984). Stimulation of growth of the chick embryo by acute hyperoxia. Respir. Physiol..

[bib0053] Stock M.K., Metcalfe J. (1987). Modulation of growth and metabolism of the chick embryo by a brief (72-hr) change in oxygen availability. J. Exp. Zool. Suppl..

[bib0054] Suarez M.E., Wilson H.R., Mather F.B., Wilcox C.J., Mc Pherson B.N. (1997). Effect of strain and age of the broiler breeder female on incubation time and chick weight. Poult. Sci..

[bib0055] Taylor L.W., Kreutziger G.O., Abercrombie G.L. (1971). The gaseous environment of the chick embryo in relation to its development and hatchability. 5. Effects of carbon dioxide and oxygen levels during the terminal days of incubation. Poult. Sci..

[bib0056] Tona K., Everaert N., Willemsen H., Gbeassor M., Decuypere E., Buyse J. (2013). Effects of interaction of incubator CO_2_ levels and mixing hatching eggs of different embryo growth trajectory on embryo physiological and hatching parameters. Brit. Poult. Sci..

[bib0057] Tona K., Onagbesan O., Bruggeman V., De Smit L., Figueiredo D., Decuypere E. (2007). Non-ventilation during early incubation in combination with dexamethasone administration during lateincubation. 1. Effects on physiological hormone levels, incubation duration and hatching events. Domest. Anim. Endocrinol..

[bib0058] Tong Q., Mc Gonnell M., Romanini C.E.B., Bergoug H., Roulston N., Exadaktylos V., Berckmans D., Bahr C., Guinebretiere M., Eterradossi N., Garain P., Demmers T. (2015). Higher levels of CO_2_ during late incubation alter the hatch time of chicken embryos. Br. Poult. Sci..

[bib0059] Tullett S.G., Deeming D.C. (1982). The relationship between eggshell porosity and oxygen consumption in the domestic fowl. Comp. Biochem. Physiol..

[bib0060] Tullett, S. 2009. Hatchery. Ross Tech: Investigating Hatchery Practice. Aviagen Inc., Scotland, UK.

[bib0061] Tullett, S. 2013. Incubating eggs at high altitude. Ross Technote: 0713-AVNR-027. Aviagen Inc., Scotland, UK.

[bib0062] Visschedijk A.H.J. (1968). The air space and embryonic respiration 3. The balance between oxygen and carbondioxide in the air space of the incubating chicken egg and its role in stimulating pipping. Brit. Poult. Sci..

[bib0063] Visschedijk A.H. (1991). Gordon memorial lecture: physics and physiology of incubation. Brit. Poult. Sci..

