# Supplementary material for: Effects of incubator oxygen and carbon dioxide concentrations on hatchability of fertile eggs, some blood parameters, and histopathological changes of broilers with different parental stock ages in high altitude
Source: Poult Sci. 2021 Nov 19;101(2):101609. doi: 10.1016/j.psj.2021.101609 (PMC8703081; doi:10.1016/j.psj.2021.101609)
Supplement: Supplementary file 1 [file mmc1.pdf]

ABANT İZZET BAYSAL ÜNİVERSİTESİ

Hayvan Araştırmaları Yerel Etik Kurulu

Sayı : 32 04./6./2018

Konu: Kararlar

BAŞVURU BİLGİLERİ (APPLICATION INFORMATION)		
	**ARAŞTIRMANIN ADI (TITLE OF THE PROJECT)**	Damızlık Yaşı ile Kuluçka Makinesindeki Karbondioksit ve Oksijen Seviyesinin Etlik Piliçlerde Embriyo Gelişimine etkileri..
	**SORUMLU ARAŞTIRMACI (PRINCIPALINVESTIGATER)**	Dr.Öğr.Üyesi Nezih OKUR
	**DİĞER ARAŞTIRMACILAR (OTHER INVESTIGATERS)**	Dr.Öğr.Üyesi S.Arda ERATALAR, Prof.Dr.Şule Yurdagül ÖZSOY, Prof.Dr.Ayşe Arzu YİĞİT, Dr.Öğr.Üyesi Ruhi KABAKÇI, Dr.Tuncer KUTLU
	**ARAŞTIRMA MERKEZİ (RESEARCH CENTER)**	AİBÜ Deney Hayvanlar Uygulama ve Araştırma Merkezi

KARAR (DECISION)		
	**Karar no (Decision No):2018/20**	**Tarih (Date): 16.05.2018**
	Dr.Öğr.Üyesi Nezih OKUR ,un sorumluluğunda yapılması tasarlanan ve yukarıda başvuru bilgileri verilen araştırma dosyası ve ilgili belgelerin incelenmesi sonucunda araştırmanın 72 adet civciv ile gerçekleştirilmesinde etik yönden sakınca olmadığına mevcudun oy birliği/oy çokluğu ile karar verilmiştir.	

**Üyeler**	**Uzmanlık Alanı**	**Kurumu**	**İmza**
Prof. Dr. Erol AYAZ (Başkan Yrd.)	Tıbbi Parazitoloji AD Öğretim Üyesi	AİBÜ Tıp Fakültesi	
Prof. Dr. Hamit COŞKUN (Üye)	Psikoloji Bölümü Öğretim Üyesi	AİBÜ Fen Edebiyat Fakültesi	
Prof. Dr. Neriman ŞENGÜL (İnvivo Üye)	Genel Cer. AD Öğretim Üyesi	AİBÜ Tıp Fakültesi	
Prof.Dr. Dr. Ali Rıza GEZİCİ (Üye)	Beyin Cer. AD Öğretim Üyesi	AİBÜ Tıp Fakültesi	
Doç. Dr. Fatih ULAŞ (Üye)	Göz Hastalıkları AD Öğretim Üyesi	AİBÜ Tıp Fakültesi	
Doç. Dr. Mustafa ŞİT (Üye)	Genel Cer. AD Öğretim Üyesi	AİBÜ Tıp Fakültesi	
Doç. Dr. Orçun TOPTAŞ (Üye)	Cerrahi AD Öğretim Üyesi	AİBÜ Diş Hek. Fakültesi	
Doç..Dr. Yeşim YENER (Üye)	AİBÜ Temel Eğitim Bölümü Öğretim Üyesi	AİBÜ Eğitim Fakültesi	
Doç. Dr. Eray KEMAHLI (Üye)	Üroloji AD Öğretim. Üyesi	AİBÜ Tıp Fakültesi	
Dr.Öğr.Üyesi Serdar GÖZÜTOK (Üye)	Yaban Hayatı ve Ekolojisi Bölümü Öğretim Üyesi	AİBÜ Ziraat ve Doğa Bilimleri Fakültesi	
Dr. Öğr. Üyesi Hayriye ORALLAR (Üye)	Kanatlı Hayvan Yetiştiriciliği Bölümü	AİBÜ Ziraat ve Doğa Bilimleri Fakültesi	
Dr. Öğr. Üyesi Ayhan ÇETİNKAYA (Üye)	Fizyoloji AD Öğretim Üyesi	AİBÜ Tıp Fakültesi	
Vetr. Hek. Enes EĞİLMEZ (Sorumlu Veteriner Hekim)	Veteriner Hekim	AİBÜ Deney Hayvanları Uygulama. ve Araştırma Merkezi	
Vet. Hek. Orhan BULUT (TC Üyesi)	Veteriner Hekim	BOLU BEYPİLİÇ AŞ.	
Av.Cihan YAVUZ (TC Üyesi)	Avukat	Tabaklar mah. İzzet Baysal cad. No:6 BOLU	
